# Transcriptional Modulation of Infertility-Associated Genes Following *Chlamydia trachomatis* Infection in Human Fallopian Tube Mesenchymal Cells: In Silico Study

**DOI:** 10.3390/genes17030302

**Published:** 2026-03-01

**Authors:** Rafaela Rodrigues, Carlos Sousa, Nuno Vale

**Affiliations:** 1 PerMed Research Group, RISE-Health, Faculty of Medicine, University of Porto, Alameda Professor Hernâni Monteiro, 4200-319 Porto, Portugal; up201908616@edu.med.up.pt (R.R.); carlos.sousa@synlab.pt (C.S.); 2 Molecular Biology Department, SYNLAB Portugal, Rua Manuel Pinto de Azevedo, 401, 4100-321 Porto, Portugal; 3 RISE-Health, Department of Community Medicine, Health Information and Decision (MEDCIDS), Faculty of Medicine, University of Porto, Rua Doutor Plácido da Costa, 4200-450 Porto, Portugal; 4 Laboratory of Personalized Medicine, Department of Community Medicine, Health Information and Decision (MEDCIDS), Faculty of Medicine, University of Porto, Rua Doutor Plácido da Costa, 4200-450 Porto, Portugal

**Keywords:** sexually transmitted infections, *Chlamydia trachomatis*, female infertility, fallopian tube, gene expression, in silico analyses

## Abstract

**Background/Objectives:** *Chlamydia trachomatis* (CT) infection is one of the most prevalent sexually transmitted infections (STIs) worldwide and has been consistently associated with adverse reproductive outcomes, including female infertility. However, the molecular mechanisms underlying this association remain incompletely understood. This study aimed to investigate whether genes previously associated with female infertility display altered expression patterns in response to CT infection by reanalyzing publicly available transcriptomic data derived from a human in vitro infection model. **Methods**: An integrative in silico approach was employed. A curated list of 106 genes associated with female infertility was compiled from publicly available databases and integrated with transcriptomic data from the Gene Expression Omnibus (GEO) dataset GSE109428, which profiles primary human fallopian tube mesenchymal cells infected in vitro with CT serovar L2. Gene expression changes were evaluated at two time points (24 and 48 h post-infection) by comparing infected cells with uninfected control samples, followed by functional and phenotype enrichment analyses. **Results**: One female infertility-associated gene (*AKAP12*) was consistently dysregulated at both 24 and 48 h post-infection. In addition, fourteen genes (*ANAPC4*, *BMP1*, *BNC2*, *BTG4*, *EFHD1*, *FBXO43*, *INHBB*, *PATL2*, *SCARB1*, *SND1*, *SYNE1*, *TRIP13*, *TTC28*, and *TUBA1C*) became significantly dysregulated exclusively at 48 h post-infection, indicating a time-dependent host transcriptional response to CT infection. Functional and phenotype enrichment analyses revealed associations with biological processes related to embryonic development and meiosis, as well as phenotypes linked to female infertility. These enriched terms were supported by a small subset of genes and were therefore interpreted cautiously. **Conclusions:** Overall, these findings suggest that CT infection modulates the expression of several infertility-associated genes and may influence biological pathways critical for female reproductive function. While exploratory, this study provides a molecular context that aligns with previously reported associations between CT infection and female infertility.

## 1. Introduction

*Chlamydia trachomatis* (CT) is one of the most prevalent bacterial sexually transmitted infections (STIs) worldwide and, despite being easily treatable with antibiotics, it remains a major public health concern, particularly among women of reproductive age [1,2,3]. Acute infections are frequently asymptomatic, which contributes to delayed diagnosis and allows persistent or recurrent infections to develop [4,5]. Subsequently, these chronic infections can lead to severe reproductive tract complications, including pelvic inflammatory disease (PID), tubal damage, ectopic pregnancy, and infertility [6,7,8,9]. The fallopian tube is one of the most affected organs, as CT infection can trigger an exacerbated inflammatory response and tissue remodeling processes that compromise the normal reproductive function [10,11]. The resulting inflammation, scarring, and fibrosis may ultimately lead to tubal occlusion, a hallmark of tubal factor infertility [12,13]. Mechanistically, CT infection usually ascends from the lower genital tract, migrating from the cervix to the endometrium and subsequently reaching the fallopian tubes, where it may cause acute or chronic PID [9,13]. Moreover, there is evidence pointing out that having a PID history is strongly associated with tubal factor infertility, characterized by partial or complete obstruction of the fallopian tubes, one of the most common causes of female infertility [14,15,16]. Although the clinical association between CT infection, PID, and tubal infertility has been recognized, the molecular mechanisms underlying the association between CT infection and long-term reproductive dysfunction are still unclear [17,18].

**Figure 1:**
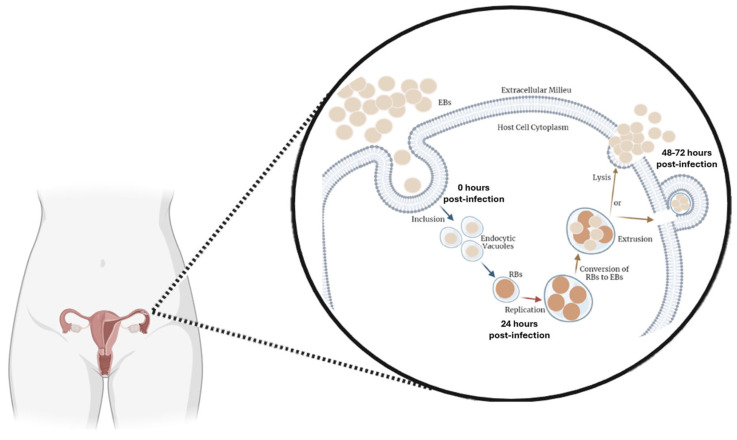
The developmental cycle of CT in the female reproductive tract. The schematic depicts the biphasic life cycle of the bacterium in the fallopian tubes. On the left, it shows in detail the transition from EBs to RBs within a membrane-bound inclusion following host cell entry. Then, RBs replicate multiple times until 24 h post-infection, and subsequently, RBs re-differentiate into EBs. Finally, there are two possible mechanisms for the extracellular EBs release: (1) lysis of the host cell or (2) extrusion, to infect other cells. The intracellular life cycle usually requires 48 to 72 h to complete. Abbreviations: EBs, elementary bodies; RBs, reticulate bodies. Figure created using BioRender—https://www.biorender.com/ (accessed on 20 January 2026).

Regarding the bacterium, CT is an obligatory intracellular pathogen with a unique biphasic developmental life cycle, alternating between two different forms, the infectious elementary body (EB) and the replicative reticulate body (RB) [19,20]. Its developmental cycle starts with the EBs entering the host cells, followed by differentiation into RBs within a membrane-bound inclusion, where they actively replicate. Subsequently, RBs re-differentiate into EBs, which are then released from the host cell to infect adjacent cells. This life cycle typically spans approximately 48–72 h, depending on host cell and environmental factors [8,20,21]. A schematic overview of the intracellular developmental cycle of CT, highlighting the temporal progression, is shown in Figure 1. These temporal stages provide a biological framework for interpreting host transcriptional responses at early (24 h) and later (48 h) time points following infection.

The temporal progression of this bacterium’s life cycle is accompanied by increasing host–pathogen interactions, inflammatory signaling, and cellular stress. Thus, as bacterial replication increases over time, more pronounced transcriptional perturbations in host cells are expected [22]. Therefore, understanding the temporal dynamics of host gene expression changes during CT infection is critical to elucidate how the bacterium may contribute to long-term reproductive dysfunction [22,23]. Indeed, chronic infection and exacerbated inflammatory responses in the female reproductive tract have been increasingly recognized as important modulators of local gene expression critical for reproductive function [9,24]. Inflammatory signaling, oxidative stress, and tissue remodeling can alter transcriptional networks leading to cell proliferation, apoptosis, extracellular matrix organization, and endocrine responsiveness, thereby creating a hostile microenvironment for gamete transport and early embryonic development and contributing to reproductive tract pathology [25,26,27,28]. In this context, CT infection has been shown to induce transcriptional reprogramming in infected reproductive tract cells, affecting not only immune and inflammatory pathways, but also genes involved in mitochondrial function, cell cycle regulation, tissue repair, and cellular stress [29,30]. These transcriptional alterations induced by CT infection may therefore contribute to long-term reproductive dysfunction by modulating genes with established roles in female fertility. In fact, female fertility is a highly coordinated biological process that depends on the integrity of multiple molecular pathways governing oocyte maturation, meiotic progression, fertilization, and early embryonic development [31,32]. Additionally, the tubal microenvironment is a critical determinant of reproductive success. The fallopian tube provides essential biochemical and paracrine support for oocyte transport, fertilization, and early embryo development [33,34]. Thus, infection-induced gene expression changes in fallopian tube cells may contribute to infertility not only through structural damage and scarring but also by disrupting the molecular microenvironment required for successful fertilization and early stages of embryogenesis [35,36].

In this context, investigating whether genes implicated in female infertility are transcriptionally modulated in response to infectious and inflammatory insults, such as CT infection, represents a biologically plausible strategy for uncovering molecular links between infection, host responses, and long-term reproductive dysfunction in females.

Thus, as the fallopian tube plays a central role in the reproductive tract pathology associated with the bacterium, this tissue represents a relevant model for investigating the molecular links between infection and infertility [9,10]. Mesenchymal cells of the fallopian tube represent a biologically relevant cellular population for studying infection-induced transcriptional changes relevant to tubal pathology. These cells contribute to extracellular matrix deposition, fibrotic remodeling, and the orchestration of inflammatory responses during tissue injury and repair [37]. In the context of CT infection, mesenchymal cells are likely to participate in the development of chronic inflammation and fibrosis that underlie tubal scarring and occlusion. Therefore, analyzing transcriptional responses in fallopian tube mesenchymal cells provides mechanistic insight into the molecular pathways linking CT infection to long-term structural and functional injury of the fallopian tubes.

Importantly, several transcriptomic studies have characterized host gene expression changes in response to CT infection in epithelial and reproductive tract cells, and the analyses have predominantly focused on immune and inflammatory signaling pathways [38,39]. Accordingly, the potential impact of CT infection on the expression of genes previously implicated in female infertility has not been systematically explored, particularly in human-based models such as fallopian tube mesenchymal cells. Moreover, the integration of infection-induced transcriptional responses with curated sets of infertility-associated genes remains largely unexplored. Addressing this gap is essential to elucidate potential molecular links between CT infection, host transcriptional dysregulation, and long-term female reproductive consequences, such as infertility. In this study, we aimed to determine whether genes previously reported to be associated with female infertility are transcriptionally modulated in response to CT infection. To address this question, we performed a knowledge-based integrative in silico analysis using publicly available transcriptomic data from human fallopian tube cells infected in vitro with CT. This in silico study design enables the efficient integration and re-analysis of existing high-throughput datasets, providing a cost- and time-effective strategy to maximize the use of publicly available resources and generate biologically meaningful hypotheses. Importantly, such analyses can highlight candidate genes and pathways for later experimental validation, thereby reducing the need for extensive exploratory in vitro studies.

By integrating publicly available transcriptomic datasets with existing gene–disease databases, this knowledge-based approach allows the systematic identification of candidate genes and pathways linking infection-induced transcriptional alterations to clinically relevant phenotypes [40,41,42]. Importantly, the prioritization of infertility-associated genes modulated during CT infection may facilitate the identification of molecular targets for future experimental validation and therapeutic intervention [43,44,45]. Thus, this study seeks to provide molecular insight into the mechanisms linking CT infection to infertility.

## 2. Materials and Methods

### 2.1. Genes and Transcriptomic Data Search Strategy

The study started with the identification of genes associated with female infertility through manual searches of publicly available genetic and genomic databases, including OMIM [46], ClinVar (NCBI) [47], and GWAS Catalog (EMBL-EBI) [48]. By applying the search terms “tubal factor infertility” and “female infertility”, genes reported to be associated with infertility were retrieved based on Mendelian inheritance, clinical genetic evidence, or genome-wide association studies. These genes were then combined into a curated, non-redundant list following manual curation.

In a second phase, transcriptomic datasets related to CT infection were searched in the Gene Expression Omnibus (GEO) database (NCBI) [49,50]. Two independent search strategies were applied: Search #1 used the terms “tubal factor infertility” AND “Chlamydia trachomatis”, and Search #2 used “fallopian tube” AND “Chlamydia trachomatis”. Search #1 returned two results, while Search #2 returned 65 results. These studies were subsequently filtered according to the following inclusion criteria: Homo Sapiens origin, publication date from 2020 onwards, and study type “Expression profiling by array”.

### 2.2. Differential Gene Expression Analysis and Integration Analysis

Differential gene expression analysis was performed using a reproducible pipeline implemented in RStudio software (version 2025.09.1+401) based on the Linear Models for Microarray Data (limma) Bioconductor package. Raw expression data for GSE109428 were retrieved using the GEOquery package. Data quality was assessed prior to and after normalization using boxplots, density plots, and principal component analysis (PCA) to evaluate sample distribution and clustering patterns (Supplementary File). Normalization between arrays was performed using quantile normalization as implemented in limma. The experimental design matrix was explicitly defined to model the three experimental conditions (24, 48 h post-infection, and uninfected controls), and contrasts were defined to compare CT-infected samples at 24 and 48 h post-infection against uninfected controls. Linear models were fitted using lmFit, followed by empirical Bayes moderation using eBayes, with an explicit design matrix and contrasts comparing both time points with the control. Multiple testing correction was performed using the Benjamini–Hochberg false discovery rate (FDR) method. Microarray probe identifiers were mapped to official gene symbols using the corresponding platform annotation file (GPL21272, Agilent microarray) retrieved from GEO [51]. Probes without valid gene symbol annotations were excluded. Moreover, when multiple probes mapped to the same gene, probe-level results were collapsed to gene level by retaining the probe with the highest absolute log2 fold change (|log2FC|) per gene. Batch correction was not applied because no batch-related covariates were present in the GEO metadata. Differentially expressed genes (DEGs) were filtered using an adjusted *p*-value < 0.05 and an |log2FC| ≥ 0.5. The resulting DEG lists were intersected with the curated list of female infertility-associated genes to identify genes modulated by CT infection for downstream functional enrichment and protein–protein interaction (PPI) analyses. All data processing, statistical analysis, integration, and visualization steps were performed in R. The full R script used for data processing and differential expression analysis is provided as Supplementary File S1.

### 2.3. Functional Enrichment Analysis

Functional enrichment analysis was performed to explore the biological relevance of infertility-associated genes whose expression was modulated by CT infection. The overlapping gene set obtained from the integration analysis was analyzed using the g:Profiler web tool [52]. Enrichment was conducted using a custom background universe consisting of all transcripts tested in the differential expression analysis (*n* = 32,063), ensuring an unbiased statistical framework. Gene Ontology (GO) Biological Process (GO:BP) terms andHuman Phenotype Ontology (HPO) terms were evaluated, and multiple testing correction was applied using the g:SCS method implemented in g:Profiler. Terms with an adjusted *p*-value < 0.05 were considered statistically significant. Enrichment results were used to support the biological interpretation of CT-induced transcriptional changes in the context of female infertility.

### 2.4. Protein–Protein Interaction (PPI) Analysis

PPI analysis was performed using the Search Tool for the Retrieval of Interacting Genes/Proteins (STRING) database (version 12.0) [53] to explore potential functional relationships among infertility-associated genes modulated by CT infection. The analysis was restricted to *Homo sapiens*, using a medium confidence threshold (0.4) with all evidence channels enabled. The resulting PPI network was visualized to assess interaction patterns, network connectivity, and the presence of potential functional modules.

### 2.5. Data Processing and Visualization

All data processing, differential expression analysis, integration steps, statistical filtering, and graphical visualizations were performed entirely in RStudio (R version 2025.09.1+401). Raw expression matrices and sample annotation files from GSE109428 were imported into R and processed using the limma package for background correction, normalization, and linear modeling. Differential expression statistics (log2FC, *p*-values, and Benjamini–Hochberg adjusted *p*-values) were computed directly in R without the use of GEO2R, which was just used before for an exploratory overview. Downstream analyses, including gene list integration, construction of the custom gene universe, functional enrichment filtering, and preparation of figures and tables, were performed using dplyr, tidyr, ggplot2, and pheatmap in RStudio. All tables and figures presented in the Results Section were generated in RStudio, and the script was available in the Supplementary File S1.

### 2.6. Independent Dataset Validation

To explore whether the prioritized infertility-associated genes showed consistent transcriptional patterns in an independent dataset, we analyzed GSE114556, a publicly available dataset of HeLa cells infected by CT for 33 h. Raw data were processed using the same normalization and differential expression pipeline described above. This analysis was performed solely for exploratory validation and is presented in the Supplementary File S2.

## 3. Results

### 3.1. Identification of Female Infertility-Associated Genes and Selection of the Transcriptomic Dataset

A total of 106 genes associated with female infertility were identified following the gene selection and curation process described above. This final curated gene list was used for subsequent integration with transcriptomic data related to CT infection. In parallel, transcriptomic datasets were selected based on the predefined inclusion criteria. After applying the filters (“fallopian tube”[All Fields] AND “Chlamydia trachomatis”[All Fields]) AND “Homo sapiens”[porgn] AND (“Expression profiling by array”[Filter] AND (“2020/01/01”[PDAT]: “2025/11/31”[PDAT]), a single dataset met all eligibility criteria. Thus, this dataset, GSE109428, entitled “Primary mesenchymal cells from human fallopian tube infected with Chlamydia trachomatis”, from da Costa AT, Mollenkopf H, Meyer TF, and Berger H, was selected for downstream analyses (Table 1) [54].

### 3.2. Integration of Infertility-Associated Genes with CT-Induced Transcriptional Changes

To identify infertility-associated genes modulated by CT infection, the lists of differentially expressed genes (DEGs) obtained at 24 and 48 h post-infection were intersected with the curated list of 106 infertility-related genes.

Of the 106 female infertility-associated genes curated in this study, we verified that only 81 were represented on the microarray platform (GPL21272) used in the selected dataset GSE109428 and could therefore be evaluated in the transcriptomic analysis. The remaining 25 genes were not represented on the platform and were thus excluded from further analysis. Of note, the list of infertility-associated genes represented and not represented on the microarray platform is provided in Table 2.

At 24 h post-infection, only one infertility-associated gene, AKAP12, was found to be significantly dysregulated and was upregulated in CT-infected cells compared with uninfected controls.

At 48 h post-infection, a total of fifteen infertility-associated genes were significantly dysregulated, indicating a broader transcriptional response at the later stage of infection. Among these, five genes (*AKAP12*, *BTG4*, *INHBB*, *PATL2*, and *SYNE1*) were upregulated, whereas ten genes (*ANAPC4*, *BMP1*, *BNC2*, *EFHD1*, *FBXO43*, *SCARB1*, *SND1*, *TRIP13*, *TTC28*, and *TUBA1C*) were downregulated. Intersection analysis across time points revealed *AKAP12* as the only infertility-associated gene consistently dysregulated at both 24 and 48 h post-infection, with concordant upregulation at both time points. Overall, a greater number of infertility-associated genes were affected at 48 h compared with 24 h post-infection. Detailed information on (log2FC) and adjusted *p*-values (adjP) for all differentially expressed infertility-associated genes is provided in Table 3. Of note, positive log2FC values indicate upregulation, whereas negative values indicate downregulation in CT-infected cells compared with uninfected controls.

**Figure 2:**
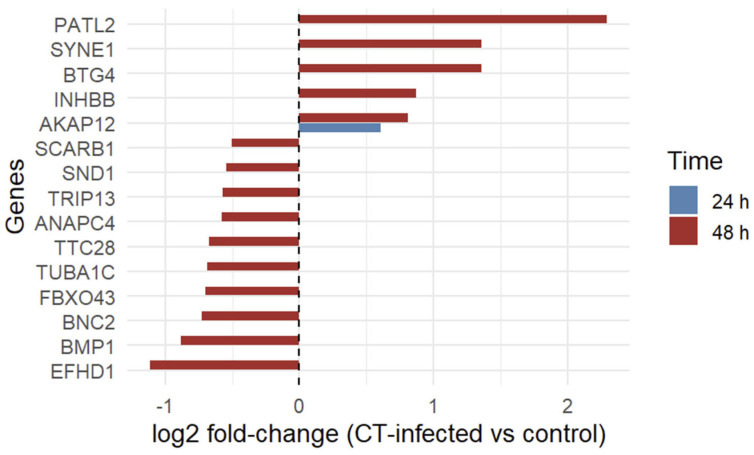
Differential expression of infertility-associated genes in human fallopian tube cells following CT infection at 24 and 48 h post-infection, shown in blue and red, respectively. Bars represent log_2_ fold change (CT-infected vs uninfected controls) for genes significantly dysregulated at one or both time points. Positive log2FC values indicate upregulation, whereas negative values indicate downregulation. Only genes significant at both time points are shown in paired comparison. Abbreviations: CT, *Chlamydia trachomatis*.

To facilitate comparison of transcriptional changes across time points, the log2*FC* of infertility-associated genes significantly dysregulated at both 24 and 48 h post-infection were visualized in a bar plot (Figure 2), highlighting the consistency in direction and magnitude of gene expression changes over both time points.

**Figure 3:**
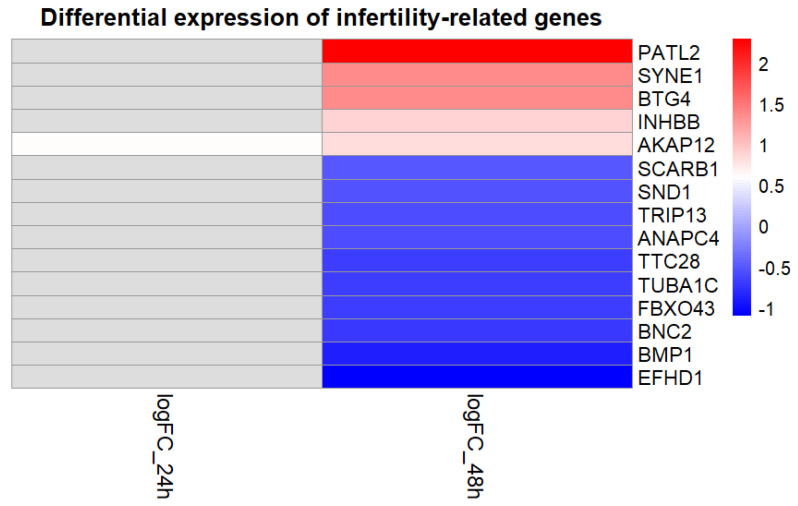
Heatmap representation of log2FC for infertility-associated genes modulated by CT infection at 24 and 48 h post-infection. Rows correspond to genes and columns to time points. Red indicates upregulation and blue indicates downregulation relative to uninfected controls, while gray cells denote genes not significantly dysregulated at the corresponding time point. Abbreviations: log2FC, log2 fold change.

Additionally, a heatmap visualization was used to summarize expression patterns of all differentially expressed infertility-associated genes across time points, illustrating both shared and time-specific transcriptional responses to CT infection (Figure 3).

### 3.3. Functional Enrichment Analysis

Functional enrichment analysis was performed using g:Profiler with a custom background universe consisting of all transcripts tested in the differential expression analysis (*n* = 32,063), which we constructed in R. Among the 15 CT-modulated infertility-associated genes, several Human Phenotype Ontology (HPO) terms showed significant enrichment, predominantly related to embryonic development, female infertility, and meiosis. These terms were driven by well-established infertility genes such as *BTG4*, *FBXO43*, *PATL2*, *and TRIP13.* Given the small size of the gene set and the fact that several enriched terms were supported by only two or three genes, the results were interpreted cautiously, and only biologically coherent terms were considered relevant. The enriched HPO terms, together with their adjusted *p*-values, effect sizes, term sizes, and gene intersections, are summarized in Table 4.

**Figure 4:**
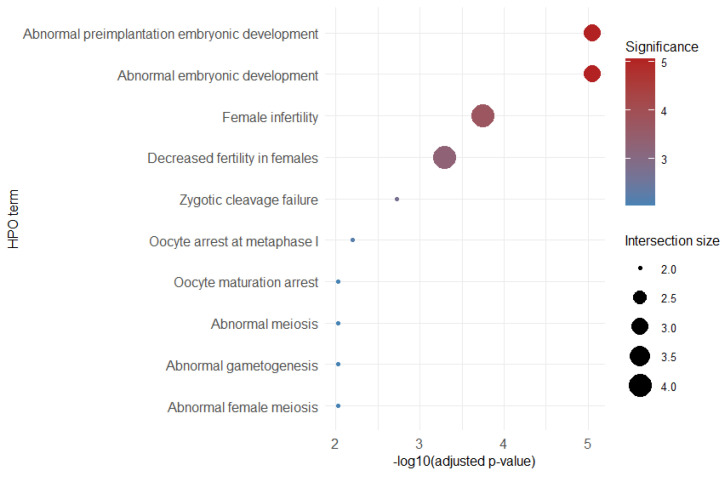
Dot plot of HPO enrichment for CT-modulated infertility-associated genes. Enrichment analysis was performed using g:Profiler with a custom background universe (n = 32,063). Only terms with adjusted *p-value* < 0.05 and intersection size ≥ 2 were included. Dot size represents the number of genes overlapping with each term, and dot color indicates the −log10 adjusted *p*-value. Biologically irrelevant or single-gene terms were excluded to avoid over-interpretation. Abbreviations: HPO—Human Phenotype Ontology.

To facilitate visualization of the enrichment patterns, we generated a dot plot summarizing the significant HPO terms identified (Figure 4).

The plot highlights the predominance of phenotypes related to abnormal embryonic development and female infertility, driven mainly by *BTG4*, *FBXO43*, *PATL2*, and *TRIP13*. Only terms with intersection size ≥ 2 and adjusted *p*-value < 0.05 were included, and biologically irrelevant or single-gene terms were excluded to avoid over-interpretation. This visualization complements Table 3 by illustrating the relative significance and gene overlap of each enriched phenotype.

### 3.4. PPI Network Analysis

**Figure 5:**
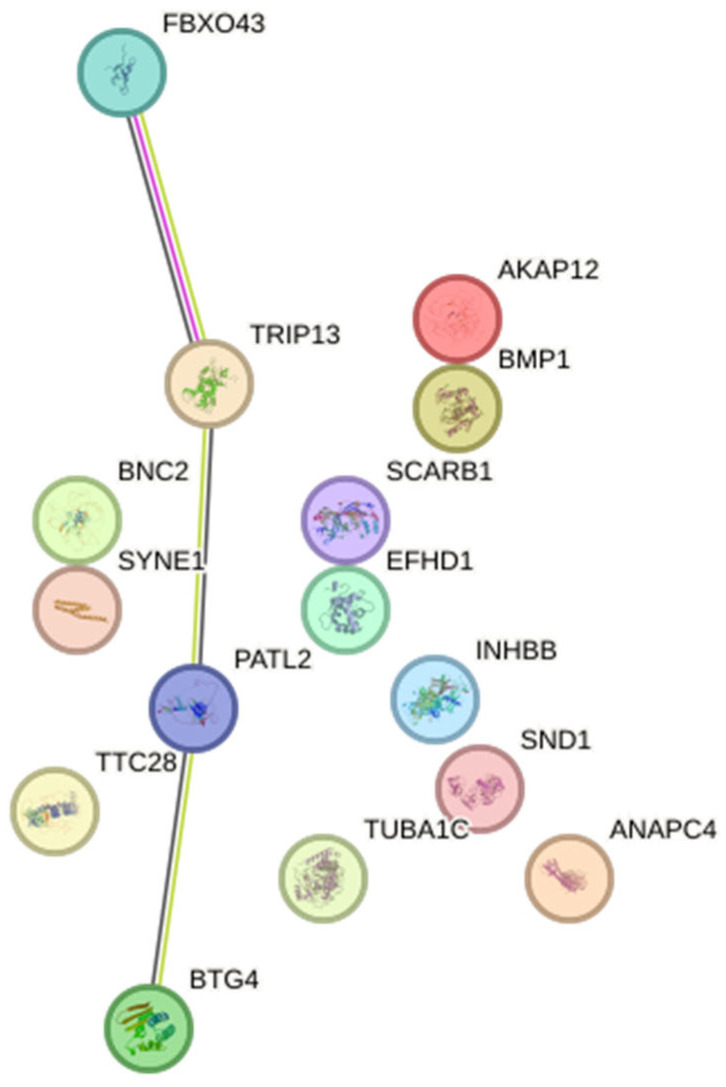
PPI network of infertility-associated genes modulated by CT infection. The network was generated using the STRING database (version 12.0; *Homo sapiens*). Nodes represent proteins and edges indicate known or predicted associations. Most connections are supported predominantly by text-mining and weak co-expression evidence, with only the FBXO43–TRIP13 interaction showing low experimental support at the applied confidence threshold (0.4). The network therefore reflects literature- and co-expression-based associations rather than robust, experimentally validated protein–protein interactions. Figure created using STRING—https://string-db.org/ (accessed on 11 February 2026).

PPI analysis revealed a sparsely connected network among the 15 infertility-associated genes modulated by CT infection (Figure 5). Also, a quantitative summary table of evidence channels for each STRING edge is provided in Appendix A.

Most edges were supported predominantly by text-mining and weak co-expression evidence, with only a single interaction (FBXO43–TRIP13) displaying low experimental support at the applied confidence threshold (Appendix A). A small cluster involving BTG4, PATL2, TRIP13, and FBXO43 was observed; however, the limited strength of the underlying evidence indicates that these associations likely reflect the literature co-mention rather than robust functional protein–protein interactions. The remaining genes appeared as isolated nodes, consistent with either independent biological roles or the absence of experimentally validated interactions currently available in public databases. Overall, the PPI analysis provides exploratory contextual information but does not support strong mechanistic inferences.

### 3.5. Independent Dataset Validation

To strengthen the robustness of our findings, we performed an independent validation analysis using an additional CT infection transcriptomic dataset (GSE114556), comparing infected (*n* = 3) and control (*n* = 3) conditions. Among the 15 infertility-associated genes highlighted previously in the GSE109428 dataset, *FBXO43* was significantly downregulated 33 h post-infection in this independent dataset after multiple-testing correction (log_2_FC = −0.69; adj. *p* = 0.013), consistent with its downregulation in the primary dataset at 48 h post-infection. Although the remaining genes did not reach statistical significance after FDR correction, several exhibited directionally concordant expression trends of regulation, specifically, *AKAP12*, *BTG4*, *INHBB*, *TRIP13*, *EFHD1*, *BNC2*, and *BMP1*. This partial concordance is notable given the limited sample size and differences in experimental context between the two datasets. The full analysis is available in Supplementary File S2.

## 4. Discussion

CT infection is a well-established risk factor for PID [55]. PID triggers exacerbated inflammation, tissue damage, and scarring in the female reproductive tract, which can lead to blockage of the fallopian tubes, impeding fertilization and the transport of the fertilized egg to the uterus for implantation. These adverse consequences of PID can culminate in ectopic pregnancy or tubal factor infertility. However, the molecular mechanisms linking CT infection to long-term reproductive dysfunction remain incompletely understood [17,56,57].

In this study, we employed a knowledge-based integrative in silico approach to investigate whether genes previously associated with female infertility exhibit transcriptional alterations in response to CT serovar L2 infection in human fallopian tube mesenchymal cells in vitro [54]. Using bioinformatic tools, we integrated curated infertility-associated genes with publicly available transcriptomic data, and we identified a subset of genes whose expression is modulated during CT infection, providing exploratory molecular insight into host responses potentially relevant to infertility.

A key finding of this study was the identification of fifteen infertility-associated genes (*PATL2*, *EFHD1*, *BTG4*, *AKAP12*, *SYNE1*, *BMP1*, *TRIP13*, *ANAPC4*, *INHBB*, *FBXO43*, *SND1*, *SCARB1*, *TTC28*, *TUBA1C*, and *BNC2*) that were dysregulated at 48 h post-infection. Among them, *AKAP12* was consistently modulated at both 24 and 48 h post-infection, suggesting a time-dependent expansion of host transcriptional responses as infection progresses (Figure 2). This temporal pattern is consistent with the notion that prolonged or unresolved CT infection may lead to cumulative molecular disturbances contributing to reproductive tract pathology [22,58,59]. Moreover, taking into account the developmental cycle of this bacterium (Figure 1), which involves alternation between two distinct forms, EBs and RBs, it is plausible that gene expression changes become more pronounced at later stages of infection (48 h post-infection), reflecting sustained host–pathogen interactions and prolonged intracellular bacterial replication. Several of the identified genes play critical roles in oocyte maturation, zygotic cleavage failure, embryo defects, and embryo implantation. Specifically, *BTG4* [60], *PATL2* [61,62,63], *TRIP13* [64], *ANAPC4* [65], *INHBB* [66], and *FBXO43* [67], supporting the biological plausibility that their dysregulation could be relevant in a reproductive context. Additionally, evidence supporting an association between *BNC2* and reproductive traits is currently limited to non-human models (zebrafish) [68]. To date, no studies in humans have demonstrated a direct role for *BNC2* in female fertility phenotypes, emphasizing the need for further functional characterization in human models.

*TRIP13* and *FBXO43*, which formed a small interaction module in the PPI network (Figure 5), were driven mainly by text-mining and weak co-expression evidence, with minimal experimental support (Appendix A). Although this association does not represent a validated functional protein–protein interaction, both genes are involved in cell cycle control and meiotic checkpoint regulation, and their concurrent transcriptional modulation during CT infection is consistent with infection-associated perturbations of cellular programs relevant to reproductive cell cycle regulation [69,70]. Importantly, some genes identified in this study, including *SYNE1*, *SCARB1*, *AKAP12*, *BMP1*, *EFHD1*, and *SND1,* have not been functionally characterized in the context of female reproduction. Their implication is supported by genome-wide association studies rather than direct mechanistic evidence [71,72]. Therefore, the present findings should be interpreted as hypothesis-generating, highlighting candidate genes whose transcriptional modulation during CT infection warrants further investigation. Functional validation in relevant reproductive cell types and in vivo models will be required to clarify whether and how these genes contribute to female reproductive biology.

Functional and phenotype enrichment analyses yielded limited results, reflecting the small size of the prioritized gene set and the dominance of a few well-annotated fertility-related genes. Although the enriched terms were consistent with reproductive phenotypes, these findings should be interpreted cautiously and viewed as descriptive rather than mechanistic. Indeed, enrichment signals derived from small gene lists are sensitive to annotation bias and do not constitute evidence for pathway-level dysregulation.

Although the fallopian tube is not the primary site of oocyte maturation, it plays an essential role in gamete transportation, fertilization, and also the first stages of embryonic development [9,73]. Exacerbated inflammatory signaling and tissue remodeling induced by CT infection in this anatomical context may affect fertility through alterations in the molecular microenvironment required for successful reproductive function [10,12]. observed here may reflect broader host responses to infection, including cellular stress, altered extracellular matrix remodeling, and cell cycle perturbation, which may indirectly influence reproductive competence [30,74]. Alternative or complementary mechanisms, such as activation of stress response pathways, cell cycle arrest, or apoptosis, may also contribute to CT-associated infertility and should be considered [75,76].

Regarding our study, several limitations must be acknowledged. First, although we incorporated exploratory validation using an additional publicly available dataset, transcriptomic resources relevant to CT infection in human cells remain extremely scarce. As a result, the available datasets derived from in vitro infection models may not fully capture the complexity of in vivo host–pathogen interactions. Second, primary mesenchymal cells from the fallopian tube do not represent the primary target of CT infection; future studies are required from additional CT infection models to validate patterns observed in this in vitro model. Third, the gene expression changes observed do not necessarily imply direct causal effects on infertility and require experimental validation at the protein and functional levels. Additionally, although we applied a rigorous, R-based pipeline for differential expression and integration, limitations inherent to microarray platforms remain (e.g., probe-level variability, potential lack of adjustment for multiple probes mapping to the same gene in the original platform design). And finally, the enrichment and PPI analyses are exploratory and driven by a relatively small number of genes, which constrain the strength of mechanistic inferences.

Despite the study limitations, our study has several strengths that should be highlighted. The data integration was guided by a predefined, hypothesis-driven framework, in detail addressing the question of whether genes previously reported to be associated with female infertility are transcriptionally altered in human cells following CT infection. In addition, the analysis was based on transcriptomic data derived from a human cell model, increasing the biological relevance of the findings, and the availability of gene expression data at two distinct time points (24 and 48 h post-infection) allowed the assessment of temporal dynamics in host transcriptional responses to CT infection. We believe that the integrative in silico strategy employed here provides a robust and cost-effective framework to generate testable hypotheses linking CT infection to female infertility. Taken together, by prioritizing genes and pathways with established reproductive relevance, this study lays the groundwork for future mechanistic investigations aimed at elucidating how CT-induced host responses contribute to long-term reproductive dysfunction.

To further contextualize the robustness of our findings, we performed an exploratory validation using a publicly available transcriptomic dataset of CT infection in HeLa cells (GSE114556). Although this dataset differs substantially from ours in several aspects, including cell type (cervical epithelial cells), the cancer-derived nature of HeLa, infection timing (33 h post-infection), and microarray platform, to the best of our knowledge, it represents the closest independent model currently accessible. In fact, *FBXO43* was significantly downregulated in both datasets, and several additional infertility-associated genes exhibited directionally concordant expression trends. These observations suggest that some components of the transcriptional response identified in fallopian tube mesenchymal cells may extend to other reproductive tract cell types, despite biological and technical heterogeneity. However, the absence of full replication across datasets reinforces that the present work should be interpreted as hypothesis-generating. Taken together, our findings underscore a broader gap in the field, despite the clinical relevance of CT–associated infertility, transcriptomic resources derived from human cells remain extremely limited. The exploratory nature of this present work reflects not the weakness of the approach, but rather a structural scarcity of datasets that constrains mechanistic progress in this area. By integrating curated infertility-associated genes with infection-responsive transcriptional profiles, our study helps delineate molecular candidates and pathways that warrant deeper investigation. Ultimately, these results highlight the urgent need for expanded, cell-type-specific transcriptomic and functional studies to advance our understanding of how CT infection contributes to long-term reproductive dysfunction.

From a clinical and translational perspective, these findings should be viewed as hypothesis-generating rather than definitive, but they offer a conceptual basis for future personalized medicine approaches. The modulation of infertility-associated genes by CT infection suggests that, in susceptible individuals, infection may intersect with genetic or molecular backgrounds that predispose to adverse reproductive outcomes. In the long term, integrating infection-induced transcriptional signatures with infertility-associated genetic profiles could help refine risk stratification for CT-associated reproductive sequelae and guide personalized follow-up strategies [77,78,79]. However, such applications will require replication in independent datasets, validation in patient-derived samples, and mechanistic studies to clarify how CT-associated transcriptional shifts influence fertility-related molecular programs. Ultimately, combining in silico prioritization with experimental and clinical research will be essential to translate these preliminary molecular insights into actionable knowledge for reproductive health, particularly in the context of personalized medicine.

## 5. Conclusions

In summary, this integrative in silico study provides evidence that *Chlamydia trachomatis* infection is associated with time-dependent transcriptional modulation of a small subset of female infertility-associated genes in human fallopian tube mesenchymal cells. By intersecting a curated infertility gene set with CT-induced transcriptomic changes, we prioritized a concise panel of candidate genes (*PATL2*, *EFHD1*, *BTG4*, *AKAP12*, *SYNE1*, *BMP1*, *TRIP13*, *ANAPC4*, *INHBB*, *FBXO43*, *SND1*, *SCARB1*, *TTC28*, *TUBA1C*, and *BNC2*) whose dysregulation may reflect host molecular responses occurring in a tissue directly implicated in CT-associated reproductive pathology. The observation that a larger number of infertility-associated genes were dysregulated at 48 h compared with 24 h post-infection is biologically plausible and consistent with the progressive nature of intracellular CT infection and host–pathogen interactions over time. Importantly, given the limited size of the gene set and the scarcity of transcriptomic data available, the present findings should be interpreted as hypothesis-generating rather than as definitive evidence of causal mechanisms linking CT infection to infertility. The enrichment results support phenotypic consistency with reproductive processes but do not establish pathway-level mechanisms. Therefore, the prioritized genes and functional themes identified here represent testable candidates that warrant validation in independent transcriptomic datasets, additional in vitro systems, and, ultimately, in patient-derived samples.

From a translational and precision-medicine perspective, these findings motivate future studies integrating infection-induced transcriptional signatures with inter-individual genetic susceptibility to enable improved risk stratification for female CT-associated reproductive sequelae. The next step will be to assess whether combined molecular features (e.g., gene-expression signatures together with host genetic profiles) can help identify individuals who may benefit from intensified screening, closer follow-up, or earlier preventive interventions. Overall, this work provides a reproducible analytical framework and a focused set of testable hypotheses to guide future mechanistic and translational research on the molecular links between CT infection and female reproductive health.

## Figures and Tables

**Table 1:** Overview of experimental design and metadata of the GSE109428 dataset [54].

	Experimental Design and Available Metadata
GEO accession	GSE109428
Platform	GPL21272: Agilent-048908 8x60K whole genome incl V1-V2 linc BioVacSafe final 048908
Organism	Homo sapiens
Cell type	Primary mesenchymal cells from the human fallopian tube
Infection conditions	*Chlamydia trachomatis* serovar L2 vs Non-infected
Time points	24 h p.i.; 48 h p.i.
n (control)	3
n (24 h p.i.)	3
n (48 h p.i.)	3
Pairing	Not reported; samples treated as independent replicates
Batch info	Not reported in GEO
Donor info	Primary cells derived from surgical specimens of patients with benign gynecological disease; no donor-level metadata (e.g., number of donors, matching across conditions, age/sex) reported in GEO
GEO samples accessions	GSM2942799–GSM2942807
Public availability	GEO

GEO: Gene Expression Omnibus; p.i.: post-infection.

**Table 2:** Coverage of infertility-associated genes in the microarray platform GPL21272 (RStudio).

Category	Genes
Genes represented on GPL21272 (*n* = 81)	*WNT4*, *FBXO48*, *SYNE1*, *PKHD1L1*, *DENND1A*, *TTC28*, *TRHR*, *TMEM74*, *UBE2K*, *PDS5A*, *GREB1*, *AKAP12*, *SLC47A2*, *INHBB*, *TNFSF12*, *CHEK2*, *EBAG9*, *ESR1*, *ADCY2*, *UBE2U*, *EFHD1*, *SND1*, *BMP1*, *DSG4*, *CDC42*, *PADI6*, *CDC20*, *MSH4*, *ASTL*, *TUBA4A*, *CACNA2D2*, *FOXL2*, *TP63*, *GABRB1*, *TRIP13*, *MSH5*, *ZP3*, *KPNA7*, *STAG3*, *WEE2*, *MOS*, *FBXO43*, *BNC2*, *TUBB8*, *ZP1*, *PANX1*, *PGR*, *ATM*, *BTG4*, *CHEK1*, *PDE3A*, *TUBA1C*, *SCARB1*, *IRS2*, *PATL2*, *ZP2*, *SPATA22*, *PSMC3IP*, *LHB*, *NLRP2*, *NLRP5*, *PABPC1L*, *FMR1*, *BMP15*, *FIGLA*, *FSHR*, *GDF9*, *HFM1*, *MCM8*, *MCM9*, *NANOS3*, *NOBOX*, *PGRMC1*, *NR5A1*, *SYCE1*, *TLE6*, *ANAPC4*, *DNAH11*, *CCNO*, *LHCGR*, *FOXP3*
Genes not represented on GPL21272 (*n* = 25)	*DSG1-AS1*, *CDKN2B-AS1*, *REC114*, *MEIOB*, *PCO*, *KASH5*, *CYP21A2*, *MEI1*, *WDR4P2*, *TEX41*, *LINC01283*, *MIR3937*, *RNU6-300P*, *PROX1-AS1*, *CDC42-AS1*, *LINC01101*, *MAILR*, *TMEM26-AS1*, *CABCOCO1*, *FZD4-DT*, *LINC02039*, *RPSAP37*, *RNU7-62P*, *MIR129-1*, *OBI1-AS1*

**Table 3:** Differentially expressed female infertility-associated genes in human fallopian tube cells following CT infection.

Gene	log2FC (24 h)	*adjP* (24 h)	log2FC (48 h)	*adjP* (48 h)	Regulation
*ANAPC4*	NA	NA	−0.576	1.29 × 10^−2^	Downregulated
*BMP1*	NA	NA	−0.886	1.96 × 10^−2^	Downregulated
*BNC2*	NA	NA	−0.725	1.18 × 10^−2^	Downregulated
*EFHD1*	NA	NA	−1.112	3.24 × 10^−4^	Downregulated
*FBXO43*	NA	NA	−0.703	8.46 × 10^−2^	Downregulated
*SCARB1*	NA	NA	−0.503	2.72 × 10^−2^	Downregulated
*SND1*	NA	NA	−0.544	1.00 × 10^−2^	Downregulated
*TRIP13*	NA	NA	−0.569	2.94 × 10^−3^	Downregulated
*TTC28*	NA	NA	−0.674	3.78 × 10^−2^	Downregulated
*TUBA1C*	NA	NA	−0.685	3.59 × 10^−2^	Downregulated
*AKAP12*	0.603	4.25 × 10^−2^	0.809	3.65 × 10^−2^	Upregulated
*BTG4*	NA	NA	1.357	6.16 × 10^−4^	Upregulated
*INHBB*	NA	NA	0.873	4.98 × 10^−3^	Upregulated
*PATL2*	NA	NA	2.293	1.47 × 10^−4^	Upregulated
*SYNE1*	NA	NA	1.360	1.77 × 10^−3^	Upregulated

log2FC—Log_2_ fold change; *adjP*—adjusted *p*-values; NA—genes not significantly dysregulated at the corresponding time point.

**Table 4:** Functional enrichment analysis of infertility-associated genes dysregulated by CT infection using g:Profiler.

Ontology/Source	Term Name	Term ID	*adjP*	Effect Size	Term Size	Intersection Size	Genes
HPO	Abnormal embryonic development	HP:0033334	8.92 × 10^−6^	5.05	6	3	*BTG4*, *FBXO43*, *TRIP13*
HPO	Abnormal preimplantation embryonic development	HP:0033335	8.92 × 10^−6^	5.05	6	3	*BTG4*, *FBXO43*, *TRIP13*
HPO	Female infertility	HP:0008222	1.80 × 10^−4^	3.75	64	4	*BTG4*, *FBXO43*, *TRIP13, PATL2*
HPO	Decreased fertility in females	HP:0000868	5.16 × 10^−4^	3.29	83	4	*BTG4*, *FBXO43*, *TRIP13, PATL2*
HPO	Zygotic cleavage failure	HP:0033336	1.90 × 10^−3^	2.72	3	2	*BTG4*, *TRIP13*
HPO	Oocyte arrest at metaphase I	HP:0031516	6.32 × 10^−3^	2.20	5	2	*PATL2, TRIP13*
HPO	Abnormal meiosis	HP:0031515	9.47 × 10^−3^	2.02	6	2	*PATL2, TRIP13*
HPO	Abnormal gametogenesis	HP:0033337	9.47 × 10^−3^	2.02	6	2	*PATL2, TRIP13*
HPO	Abnormal female meiosis	HP:0033338	9.47 × 10^−3^	2.02	6	2	*PATL2, TRIP13*
HPO	Oocyte maturation arrest	HP:0034914	9.47 × 10^−3^	2.02	6	2	*PATL2, TRIP13*

HPO—Human Phenotype Ontology; *adjP*—adjusted *p-*values. Multiple testing correction was applied using the g:SCS method. Only terms with adjusted *p*-value < 0.05 are shown.

## Data Availability

The original contributions presented in this study are included in the article/Supplementary Material. Further inquiries can be directed to the corresponding author.

## Supplementary Materials

The following supporting information can be downloaded at: https://www.mdpi.com/article/10.3390/genes17030302/s1, Supplementary File S1: R script containing the main analysis of the primary dataset analysed; Supplementary File S2: R script containing an additional analysis using an independent, lower-power dataset.

## Abbreviations

The following abbreviations are used in this manuscript:

CT	*Chlamydia trachomatis*
EB	Elementary Body
RB	Reticulate Body
STIs	Sexually Transmitted Infections
AdjP	Adjusted *p*-values
log2FC	Log_2_ Fold Change
HPO	Human Phenotype Ontology
GO:BP	Gene Ontology–Biological Process
PPI	Protein–Protein Interaction
STRING	Search Tool for the Retrieval of Interacting Genes/Proteins
OMIM	Online Mendelian Inheritance in Man
NCBI	National Center for Biotechnology Information
EMBL	European Molecular Biology Laboratory
PCA	Principal Component Analysis
DEG	Differentially Expressed Genes
p.i.	Post-infection
GEO	Gene Expression Omnibus
PID	Pelvic Inflammatory Disease
GWAS	Genome-wide Association Studies

## Appendix A

**Table A1:** Evidence channels supporting STRING interactions among the 15 CT-modulated infertility-associated genes.

Gene Pair	Experiments	Co-Expression	Databases	Text Mining	Combined Score
*FBXO43*–*TRIP13*	0.066	0.215	0	0.295	0.438
*BTG4*–*PATL2*	0	0.067	0	0.443	0.458
*PATL2*–*TRIP13*	0	0.055	0	0.445	0.453

## References

[1] ECDC STI Cases on the Rise Across Europe. https://www.ecdc.europa.eu/en/news-events/sti-cases-rise-across-europe.

[2] Huai P., Li F., Chu T., Liu D., Liu J., Zhang F. (2020). Prevalence of genital C. trachomatis infection in the general population: A meta-analysis. BMC Infect. Dis..

[3] Rodrigues R., Marques L., Vieira-Baptista P., Sousa C., Vale N. (2022). Therapeutic Options for Chlamydia trachomatis Infection: Present and Future. Antibiotics.

[4] Tang W., Mao J., Li K.T., Walker J.S., Chou R., Fu R., Chen W., Darville T., Klausner J., Tucker J.D. (2020). Pregnancy and fertility-related adverse outcomes associated with Chlamydia trachomatis infection: A global systematic review and meta-analysis. Sex. Transm. Infect..

[5] Yao H., Li C., Tian F., Liu X., Yang S., Xiao Q., Jin Y., Huang S., Zhao P., Ma W. (2023). Evaluation of Chlamydia trachomatis screening from the perspective of health economics: A systematic review. Front. Public Health.

[6] Rodrigues R., Sousa C., Vale N. (2024). Deciphering the Puzzle: Literature Insights on C. trachomatis-Mediated Tumorigenesis, Paving the Way for Future Research. Microorganisms.

[7] Witkin Steven S., Minis E., Athanasiou A., Leizer J., Linhares Iara M.C. (2017). trachomatis: The Persistent Pathogen. Clin. Vaccine Immunol..

[8] Elwell C., Mirrashidi K., Engel J. (2016). Chlamydia cell biology and pathogenesis. Nat. Rev. Microbiol..

[9] Rodrigues R., Sousa C., Barros A., Vale N. (2025). Chlamydia trachomatis: From Urogenital Infections to the Pathway of Infertility. Genes.

[10] Han J., Sadiq N.M. (2025). Anatomy, Abdomen and Pelvis: Fallopian Tube.

[11] Hafner L.M. (2015). Pathogenesis of fallopian tube damage caused by Chlamydia trachomatis infections. Contraception.

[12] Igietseme J.U., Omosun Y., Nagy T., Stuchlik O., Reed M.S., He Q., Partin J., Joseph K., Ellerson D., George Z. (2017). Molecular Pathogenesis of Chlamydia Disease Complications: Epithelial-Mesenchymal Transition and Fibrosis. Infect. Immun..

[13] Ling H., Luo L., Dai X., Chen H. (2022). Fallopian tubal infertility: The result of Chlamydia trachomatis-induced fallopian tubal fibrosis. Mol. Cell Biochem..

[14] Callan T., Woodcock S., Huston W.M. (2021). Ascension of Chlamydia is moderated by uterine peristalsis and the neutrophil response to infection. PLoS Comput. Biol..

[15] Hunt S., Vollenhoven B. (2023). Pelvic inflammatory disease and infertility. Aust. J. Gen. Pract..

[16] Liu L., Li C., Sun X., Liu J., Zheng H., Yang B., Tang W., Wang C. (2022). Chlamydia infection, PID, and infertility: Further evidence from a case-control study in China. BMC Women’s Health.

[17] Alexiou Z.W., Hoenderboom B.M., Hoebe C.J.P.A., Dukers-Muijrers N.H.T.M., Götz H.M., van der Sande M.A.B., de Vries H.J.C., den Hartog J.E., Morré S.A., van Benthem B.H.B. (2024). Reproductive tract complication risks following Chlamydia trachomatis infections: A long-term prospective cohort study from 2008 to 2022. Lancet Reg. Health–Eur..

[18] Passos L.G., Terraciano P., Wolf N., Oliveira F.D.S., Almeida I., Passos E.P. (2022). The Correlation between Chlamydia Trachomatis and Female Infertility: A Systematic Review. Rev. Bras. Ginecol. Obstet..

[19] Rodrigues R., Sousa C., Vale N. (2022). Chlamydia trachomatis as a Current Health Problem: Challenges and Opportunities. Diagnostics.

[20] Rodrigues R., Vieira-Baptista P., Catalão C., Borrego M.J., Sousa C., Vale N. (2023). Chlamydial and Gonococcal Genital Infections: A Narrative Review. J. Pers. Med.

[21] Bugalhão J.N., Mota L.J. (2019). The multiple functions of the numerous *C. trachomatis* secreted proteins: The tip of the iceberg. Microb. Cell.

[22] Xia M., Bumgarner R., Lampe M., Stamm W. (2003). *Chlamydia trachomatis* Infection Alters Host Cell Transcription in Diverse Cellular Pathways. J. Infect. Dis..

[23] Ohmer M., Tzivelekidis T., Niedenführ N., Volceanov-Hahn L., Barth S., Vier J., Börries M., Busch H., Kook L., Biniossek M.L. (2019). Infection of HeLa cells with Chlamydia trachomatis inhibits protein synthesis and causes multiple changes to host cell pathways. Cell Microbiol..

[24] Kessler M., Hoffmann K., Fritsche K., Brinkmann V., Mollenkopf H., Thieck O., Costa A., Braicu H., Sehouli J., Mangler M. (2019). Chronic *Chlamydia* infection in human organoids increases stemness and promotes age-dependent CpG methylation. Nat. Commun..

[25] Agarwal A., Aponte-Mellado A., Premkumar B.J., Shaman A., Gupta S. (2012). The effects of oxidative stress on female reproduction: A review. Reprod. Biol. Endocrinol..

[26] Qing Q., Yaonan L., Ziqin C., Zhihui L., Ting Z., Jing Z., Yanlin Z., Song C., Ling W. (2025). Update on the pathogenesis of endometriosis-related infertility based on contemporary evidence. Front. Endocrinol..

[27] Nanda A., K T., Banerjee P., Dutta M., Wangdi T., Sharma P., Chaudhury K., Jana S.K. (2020). Cytokines, Angiogenesis, and Extracellular Matrix Degradation are Augmented by Oxidative Stress in Endometriosis. Ann. Lab. Med..

[28] Chen Y.S., Tian H.X., Rong D.C., Wang L., Chen S., Zeng J., Xu H., Mei J., Wang L.Y., Liou Y.L. (2025). ROS homeostasis in cell fate, pathophysiology, and therapeutic interventions. Mol. Biomed..

[29] Caven L., Brinkworth A., Carabeo R. (2023). Chlamydia trachomatis induces the transcriptional activity of host YAP in a Hippo-independent fashion. Front. Cell. Infect. Microbiol..

[30] Cheong H.C., Rommel M.I., Cheok Y.Y., Chan Y.T., Tang T.F., Sulaiman S., Looi C.Y., Arulanandam B., Chang L.Y., Wong W.F. (2025). Chlamydia trachomatis disrupts host metabolism in primary cervical epithelial cells. World J. Microbiol. Biotechnol..

[31] Solovova O.A., Chernykh V.B. (2022). Genetics of Oocyte Maturation Defects and Early Embryo Development Arrest. Genes.

[32] Baldini G.M., Ferri D., Malvasi A., Laganà A.S., Vimercati A., Dellino M., Baldini D., Trojano G. (2024). Genetic Abnormalities of Oocyte Maturation: Mechanisms and Clinical Implications. Int. J. Mol. Sci..

[33] Shaw J.L., Horne A.W. (2012). The paracrinology of tubal ectopic pregnancy. Mol. Cell. Endocrinol..

[34] Ezzati M., Djahanbakhch O., Arian S., Carr B.R. (2014). Tubal transport of gametes and embryos: A review of physiology and pathophysiology. J. Assist. Reprod. Genet..

[35] Mulayim N., Palter S., Selam B., Arici A. (2003). Expression and regulation of interleukin-8 in human fallopian tubal cells. Am. J. Obstet. Gynecol..

[36] Sipes J., Rayamajhi S., Bantis L.E., Madan R., Mitra A., Puri R., Rahman M.M., Ahmmed F., Pathak H., Godwin A. (2025). Spatial transcriptomic profiling of the human fallopian tube epithelium reveals region-specific gene expression patterns. Commun. Biol..

[37] Akasaka Y. (2022). The Role of Mesenchymal Stromal Cells in Tissue Repair and Fibrosis. Adv. Wound Care..

[38] Hayward R.J., Marsh J.W., Humphrys M.S., Huston W.M., Myers G.S.A. (2019). Early Transcriptional Landscapes of Chlamydia trachomatis-Infected Epithelial Cells at Single Cell Resolution. Front. Cell. Infect. Microbiol..

[39] Amaral A.F., McQueen B.E., Bellingham-Johnstun K., Poston T.B., Darville T., Nagarajan U.M., Laplante C., Käser T. (2021). Host–Pathogen Interactions of Chlamydia trachomatis in Porcine Oviduct Epithelial Cells. Pathogens.

[40] Chasman D., Walters K.B., Lopes T.J., Eisfeld A.J., Kawaoka Y., Roy S. (2016). Integrating Transcriptomic and Proteomic Data Using Predictive Regulatory Network Models of Host Response to Pathogens. PLoS Comput. Biol..

[41] Dinarvand M., Koch F.C., Al Mouiee D., Vuong K., Vijayan A., Tanzim A.F., Azad A.K.M., Penesyan A., Castaño-Rodríguez N., Vafaee F. (2022). dRNASb: A systems biology approach to decipher dynamics of host-pathogen interactions using temporal dual RNA-seq data. Microb. Genom..

[42] Zhu G.D., Cao X.J., Li Y.P., Li J.X., Leng Z.J., Xie L.M., Guo X.G. (2021). Identification of differentially expressed genes and signaling pathways in human conjunctiva and reproductive tract infected with Chlamydia trachomatis. Hum. Genomics..

[43] Lu J., Zhang W., He Y., Jiang M., Liu Z., Zhang J., Zheng L., Zhou B., Luo J., He C. (2025). Multi-omics decodes host-specific and environmental microbiome interactions in sepsis. Front. Microbiol..

[44] Pinu F.R., Beale D.J., Paten A.M., Kouremenos K., Swarup S., Schirra H.J., Wishart D. (2019). Systems Biology and Multi-Omics Integration: Viewpoints from the Metabolomics Research Community. Metabolites.

[45] Durmuş S., Çakır T., Özgür A., Guthke R. (2015). A review on computational systems biology of pathogen-host interactions. Front. Microbiol..

[46] OMIM. https://www.omim.org/.

[47] ClinVar. https://www.ncbi.nlm.nih.gov/clinvar/.

[48] GWAS Catalog. https://www.ebi.ac.uk/gwas/.

[49] Gene Expression Omnibus. https://www.ncbi.nlm.nih.gov/geo/.

[50] NCBI: GEO2R. https://www.ncbi.nlm.nih.gov/geo/geo2r/.

[51] Platform GPL21272. https://www.ncbi.nlm.nih.gov/geo/query/acc.cgi?acc=GPL21272.

[52] g:Profiler. https://biit.cs.ut.ee/gprofiler/gost.

[53] STRING. https://string-db.org/.

[54] Series GSE109428. https://www.ncbi.nlm.nih.gov/geo/query/acc.cgi?acc=GSE109428.

[55] den Heijer C.D.J., Hoebe C.J.P.A., Driessen J.H.M., Wolffs P., van den Broek I.V.F., Hoenderboom B.M., Williams R., de Vries F., Dukers-Muijrers N.H.T.M. (2019). Chlamydia trachomatis and the Risk of Pelvic Inflammatory Disease, Ectopic Pregnancy, and Female Infertility: A Retrospective Cohort Study Among Primary Care Patients. Clin. Infect. Dis..

[56] Mongane J., Hendwa E., Sengeyi D. (2024). Association between bacterial vaginosis, *Chlamydia trachomatis* infection and tubal factor infertility in Bukavu, Democratic Republic of Congo. BMC Infect. Dis..

[57] Kim J., Ślęczkowska M., Nobre B., Wieringa P. (2025). Study Models for *Chlamydia trachomatis* Infection of the Female Reproductive Tract. Microorganisms.

[58] Olagoke O., Chittaranjan S., Dean D. (2025). Transcriptional profiling of Chlamydia trachomatis and its host in an ex vivo endocervical primary cell culture system using dual RNA sequencing. Front. Cell. Infect. Microbiol..

[59] Dzakah E.E., Huang L., Xue Y., Wei S., Wang X., Chen H., Shui J., Kyei F., Rashid F., Zheng H. (2021). Host cell response and distinct gene expression profiles at different stages of Chlamydia trachomatis infection reveals stage-specific biomarkers of infection. BMC Microbiol..

[60] Zheng W., Zhou Z., Sha Q., Niu X., Sun X., Shi J., Zhao L., Zhang S., Dai J., Cai S. (2020). Homozygous Mutations in BTG4 Cause Zygotic Cleavage Failure and Female Infertility. Am. J. Hum. Genet..

[61] Cao Q., Zhao C., Wang C., Cai L., Xia M., Zhang X., Han J., Xu Y., Zhang J., Ling X. (2021). The Recurrent Mutation in PATL2 Inhibits Its Degradation Thus Causing Female Infertility Characterized by Oocyte Maturation Defect Through Regulation of the Mos-MAPK Pathway. Front. Cell Dev. Biol..

[62] Liu Z., Zhu L., Wang J., Luo G., Xi Q., Zhou X., Li Z., Yang X., Duan J., Jin L. (2020). Novel homozygous mutations in PATL2 lead to female infertility with oocyte maturation arrest. J. Assist. Reprod. Genet..

[63] Hu H.Y., Zhang G.H., Deng W.F., Wei T.Y., Feng Z.K., Li C.X., Li S.J., Liu J.E., Tian Y.P. (2024). Novel PATL2 variants cause female infertility with oocyte maturation defect. J. Assist. Reprod. Genet..

[64] Zhang Z., Li B., Fu J., Li R., Diao F., Li C., Chen B., Du J., Zhou Z., Mu J. (2020). Bi-allelic Missense Pathogenic Variants in TRIP13 Cause Female Infertility Characterized by Oocyte Maturation Arrest. Am. J. Hum. Genet..

[65] Yanez L.Z., Han J., Behr B.B., Pera R.A.R., Camarillo D.B. (2016). Human oocyte developmental potential is predicted by mechanical properties within hours after fertilization. Nat. Commun..

[66] Zhang H., Wang Z., Zhou Q., Cao Z., Jiang Y., Xu M., Liu J., Zhou J., Yan G., Sun H. (2023). Downregulated INHBB in endometrial tissue of recurrent implantation failure patients impeded decidualization through the ADCY1/cAMP signalling pathway. J. Assist. Reprod. Genet..

[67] Wang W., Wang W., Xu Y., Shi J., Fu J., Chen B., Mu J., Zhang Z., Zhao L., Lin J. (2021). FBXO43 variants in patients with female infertility characterized by early embryonic arrest. Hum. Reprod..

[68] Lang M.R., Patterson L.B., Gordon T.N., Johnson S.L., Parichy D.M. (2009). Basonuclin-2 requirements for zebrafish adult pigment pattern development and female fertility. PLoS Genet..

[69] Zhang Q., Dong Y., Hao S., Tong Y., Luo Q., Aerxiding P. (2019). The oncogenic role of TRIP13 in regulating proliferation, invasion, and cell cycle checkpoint in NSCLC cells. Int. J. Clin. Exp. Pathol..

[70] Wu S., Qin L., Yang J., Wang J., Shen Y. (2023). Association between F-box-only protein 43 overexpression and hepatocellular carcinoma pathogenesis and prognosis. Cancer Med..

[71] Venkatesh S.S., Wittemans L.B.L., Palmer D.S., Baya N.A., Ferreira T., Hill B., Lassen F.H., Parker M.J., Reibe S., Elhakeem A. (2025). Genome-wide analyses identify 25 infertility loci and relationships with reproductive traits across the allele frequency spectrum. Nat. Genet..

[72] Zheng X., Zhong W., O’Connell C.M., Liu Y., Haggerty C.L., Geisler W.M., Anyalechi G.E., Kirkcaldy R.D., Wiesenfeld H.C., Hillier S.L. (2021). Host Genetic Risk Factors for Chlamydia trachomatis-Related Infertility in Women. J. Infect. Dis..

[73] Barrière P., Thibault E., Jean M. (2002). Role of Fallopian tube in fertilization. Rev. Prat..

[74] Steiert B., Faris R., Weber M. (2023). In Search of a Mechanistic Link between Chlamydia trachomatis-Induced Cellular Pathophysiology and Oncogenesis. Infect. Immun..

[75] Luo F., Wen Y., Zhao L., Su S., Lei W., Chen L., Chen C., Huang Q., Li Z. (2022). LncRNA ZEB1-AS1/miR-1224-5p/MAP4K4 axis regulates mitochondria-mediated HeLa cell apoptosis in persistent Chlamydia trachomatis infection. Virulence.

[76] Cheong H., Sulaiman S., Looi C., Chang L., Wong W. (2023). Chlamydia Infection Remodels Host Cell Mitochondria to Alter Energy Metabolism and Subvert Apoptosis. Microorganisms.

[77] Malogajski J., Branković I., Land J., Thomas P., Morré S., Ambrosino E. (2019). The Potential Role for Host Genetic Profiling in Screening for Chlamydia-Associated Tubal Factor Infertility (TFI)—New Perspectives. Genes.

[78] Sadeghi M.R. (2017). How Can Personalized Medicine Improve Assisted Reproduction Technology Outcomes?. J. Reprod. Infertil..

[79] Rodrigues R., Silva A.R., Sousa C., Vale N. (2025). Disrupted Cervicovaginal Microbiota: Its Role in *Chlamydia trachomatis* Genital Infection and Associated Reproductive Outcomes. Int. J. Mol. Sci..

